# Enhanced Immunohistochemistry Interpretation with a Machine Learning-Based Expert System

**DOI:** 10.3390/diagnostics14171853

**Published:** 2024-08-24

**Authors:** Anca Iulia Neagu, Diana Gina Poalelungi, Ana Fulga, Marius Neagu, Iuliu Fulga, Aurel Nechita

**Affiliations:** 1 Faculty of Medicine and Pharmacy, Dunarea de Jos University of Galati, 35 AI Cuza St., 800010 Galati, Romania; ancazanoschi@gmail.com (A.I.N.); ana.fulgaa@gmail.com (A.F.); fulgaiuliu@yahoo.com (I.F.); nechitaaurel@yahoo.com (A.N.); 2 Saint John Clinical Emergency Hospital for Children, 800487 Galati, Romania; 3 Saint Apostle Andrew Emergency County Clinical Hospital, 177 Brailei St., 800578 Galati, Romania

**Keywords:** immunohistochemistry, machine learning, prediction, diagnosis, cancer

## Abstract

Background: In recent decades, machine-learning (ML) technologies have advanced the management of high-dimensional and complex cancer data by developing reliable and user-friendly automated diagnostic tools for clinical applications. Immunohistochemistry (IHC) is an essential staining method that enables the identification of cellular origins by analyzing the expression of specific antigens within tissue samples. The aim of this study was to identify a model that could predict histopathological diagnoses based on specific immunohistochemical markers. Methods: The XGBoost learning model was applied, where the input variable (target variable) was the histopathological diagnosis and the predictors (independent variables influencing the target variable) were the immunohistochemical markers. Results: Our study demonstrated a precision rate of 85.97% within the dataset, indicating a high level of performance and suggesting that the model is generally reliable in producing accurate predictions. Conclusions: This study demonstrated the feasibility and clinical efficacy of utilizing the probabilistic decision tree algorithm to differentiate tumor diagnoses according to immunohistochemistry profiles.

## 1. Introduction

Immunohistochemistry (IHC) is a critical staining technique that facilitates the determination of cellular origins through the analysis of specific antigen expressions within tissue samples, thereby aiding in both diagnostic and prognostic assessments [1]. In addition to determining the origin of tumors, IHC is used to subtype tumors, evaluate the effectiveness of therapies, predict patient prognosis through the assessment of prognostic markers, and differentiate precancerous lesions by examining molecular alterations [2]. However, the rapidly expanding body of knowledge regarding IHC positivity in certain neoplasms often leads to divergent interpretations in standard procedures, especially in certain complex cases [3]. 

The interpretation of IHC data can vary based on the experience and expertise of individual pathologists. Researchers now have access to thousands of new antibodies and IHC-staining data across a wide range of tumor types [4]. Consequently, it is impractical for pathologists to memorize every molecular signal identified by the constantly evolving repertoire of antibodies in tumors originating from various tissues [5]. This issue can be solved by the use of standardized IHC panels and algorithmic techniques for specific diagnoses, but in practice, every case has its unique characteristics, and in certain situations, the use of IHC panels might be both time-consuming and expensive [6]. Notably, discovering a method to differentiate between various tumor subtypes would be highly desirable, particularly concerning potential clinical applications [7]. To this end, it would be highly advantageous to develop a new and practical cancer diagnostic tool that ideally uses as few distinguishing markers as possible, which can be used as an efficient and reliable method to identify and classify various tumor types. In recent decades, machine-learning (ML) technologies [8] have facilitated the management of high-dimensional and complex cancer data by creating reliable and user-friendly automated diagnostic tools for clinical applications. This advancement has allowed us to uncover new insights into the relationships between these changes and human cancers [9].

The latest advancements in ML have significantly improved the efficiency and speed of processing raw medical data. By incorporating these advanced technologies into the diagnostic process, pathologists can acquire valuable knowledge, enabling them to analyze large volumes of medical data more effectively and efficiently [10]. Deep-learning techniques, recently developed and rapidly gaining popularity, excel due to their ability to perform automated feature extraction and representation learning. As part of the learning process, this approach can autonomously extract information from images, leading to significantly improved performance in classification tasks [11]. Numerous studies have demonstrated that deep-learning systems outperform cutting-edge techniques in various medical-imaging analytic tasks, including computer-aided diagnosis, segmentation, detection, and classification [12,13,14]. 

The aim of this study was to identify a model that could predict histopathological diagnoses based on specific immunohistochemical markers.

## 2. Materials and Methods

**Figure 1:**
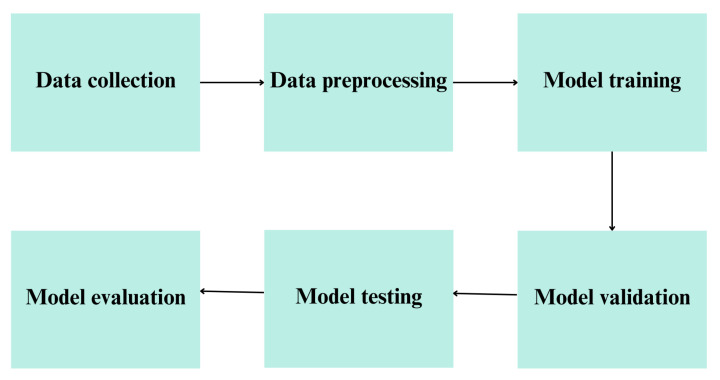
Flowchart showing the study methodology.

To provide a clear understanding of our methodology, we have included a flowchart (Figure 1) that outlines the key steps involved in developing the proposed machine-learning model for predicting histopathological diagnoses based on IHC markers.

### 2.1. Sample Data Collection and Preprocessing

In order to develop the database for the ML tool to process and analyze, we conducted a retrospective study between 2019 and 2023, collecting data from the Pathology Department of “Saint John” Hospital in Galati, Romania, after obtaining the approval of the Ethics Committee. For each case, informed consent was obtained from the patient, indicating agreement with the collection, storage, and use of biological samples for both diagnostic and scientific purposes. Any data related to patient identification, except the histopathological diagnosis and the IHC results, were blinded before data processing. The diagnoses following immunohistochemical evaluation were collected, and for each case, the specific IHC marker panels used, and their positive or negative results, were documented. 

Cases with insufficient IHC profiles—those without any markers indicating the origin of the tumor, IHC profiles with fewer than three markers, or unclear findings—were eliminated. 

After collecting the data, a total of 686 cases were included in the primary dataset. IHC antibodies, averaging 5.4 per case (range 3–11), were utilized in the diagnostic process, with a total of 58 IHC markers identified and used in various combinations in panels to support the histopathological diagnosis of each case. These markers were utilized based on their relevance to various tumor types and included common antibodies such as Cytokeratin AE1/AE3, Cytokeratin 7, Cytokeratin 20, p63, Cytokeratin 5/6, E-cadherin, Estrogen Receptor, Progesterone Receptor, p53, S100, Melan-A, Human Melanoma Black 45 (HMB45), Epithelial Membrane Antigen (EMA), CD45, CD20, CD3, CD10, CD34, Thyroid Transcription Factor 1 (TTF1), Vimentin, Actin, Calretinin, Alpha Methyl Acyl CoA Racemase (AMACR), Prostate-Specific Antigen (PSA), and Glial Fibrillary Acidic Protein (GFAP). A total of 32 histopathological diagnoses were identified, but due to the limited sample size for some diagnoses, only 20 were included in the computations performed by the ML model, with the following distribution: invasive breast carcinoma, ductal type (15.31%), squamous cell carcinoma (12.54%), prostatic acinar adenocarcinoma (9.33%), pulmonary adenocarcinoma, not otherwise specified (NOS) (8.75%), colorectal adenocarcinoma, NOS (7.43%), serous carcinoma (4.37%), endometrioid carcinoma (3.21%), melanoma (3.21%), invasive breast carcinoma, lobular type (3.06%), basal cell carcinoma (2.62%), urothelial carcinoma (2.62%), gastric adenocarcinoma, NOS (2.04%), small cell neuroendocrine carcinoma (1.89%), invasive breast cancer, other types (1.75%), colorectal mucinous adenocarcinoma (1.60%), glioblastoma (1.60%), hepatocellular carcinoma (1.46%), cholangiocarcinoma (1.46%), Kaposi sarcoma (1.46%), and undifferentiated pleomorphic sarcoma (1.46%). Diagnoses like malignant mesothelioma, mixed germ cell tumor, or non-Hodgkin lymphoma, with less than 10 cases, were categorized as “Other” by the program due to their limited numbers, representing 12.83% of the total cases.

To test the ML model, a secondary dataset comprising 21 cases from an alternative center was created to mitigate the bias associated with diagnoses conducted by the same pathologists. This new dataset adhered to the same inclusion and exclusion criteria as the primary dataset, encompassing the most frequent diagnoses, such as invasive ductal carcinoma of the breast or pulmonary adenocarcinoma, as well as diagnoses categorized as “Other”, such as pancreatic adenocarcinoma. 

All the collected data were compiled into two Excel tables with 59 columns, with the first column containing the histopathological diagnosis and the remaining columns listing the IHC markers with their respective positive or negative results, or not applicable (N/A) if the marker was not used for that specific case.

### 2.2. Machine-Learning Algorithm Model and Model Development

The ML algorithm employed in our study leverages Bayes’ theorem, a fundamental concept in probability theory and statistics that connects conditional probabilities with marginal probabilities. It states that if the pre-event probability is known, the post-event probability can be determined. Conditional probabilities indicate the likelihood of one event occurring, given that another event has occurred. Additionally, the probabilities of detecting events separately are also considered [15]. 

IHC results are binary, indicating either positive or negative outcomes, and pathologists possess practical knowledge about the probability of specific IHC markers being positive or negative in each tumor, also based on well-documented information in textbooks and other publications. The probability of positivity represents the number of positive cases out of all the diagnoses. After obtaining the test results, the likelihood of each diagnosis can be computed by multiplying the prior probability by the chance of each test being positive or negative. This allows for the comparison of post-probability values to determine which diagnosis has the highest likelihood.

To generate a prediction model, the Exploratory Desktop software, version 6.12.5.2, developed by Exploratory, Inc. was used. Exploratory Desktop offers a straightforward and user-friendly interface for accessing several data sources, transforming and cleaning data, visualizing and analyzing data, and using a wide range of machine-learning algorithms to explore data and gain deeper insights quickly. XGBoost, or eXtreme Gradient Boosting, is a well-liked machine-learning algorithm that creates prediction models in the form of an ensemble of weak prediction models—usually Decision Trees—for regression and classification tasks. Similar to previous boosting techniques, it constructs the model step-by-step and extends it by permitting optimization of any differentiable loss function. 

### 2.3. Model Training, Validation and Testing

The first step in training the model was to import our Excel dataset into the data frame from the Exploratory main interface. Once the data were loaded, the wrangling tools available were used to make some minor adjustments to eliminate any program errors, misinterpretation of data, or false results. In the second step, the XGBoost learning model was applied, where the input variable (target variable) was the histopathological diagnosis and the predictors (independent variables influencing the target variable) were the immunohistochemical markers. In the final step, the model training parameters were set; in the first instance, the default parameters provided by XGBoost were used and the model performance was checked followed by a grid search for hyperparameter tuning to find the best parameter combination.

To validate the model, the XGBoost advanced settings have an option that allows splitting the dataset into various ratios of training data and validation data, performing the training and validation at the same time. We chose a ratio of 7:3, and to avoid overfitting, we increased the number of iterations and the number of early stopping iterations. Finally, the negative log-likelihood was chosen for the multiclass classification metric. After the model was properly trained and validated, we saved it to apply the testing data set.

Testing the XGBoost model in Exploratory involved the following steps: importing the testing dataset in Exploratory, applying the trained model to the test data, and the program outputting the predicted values alongside the actual value in a new data frame. By following these detailed steps, we could effectively assess our model’s generalization ability and make informed decisions on any further tuning or adjustments.

### 2.4. Model Performance Evaluation

After the program computes the data, the micro- and macro-average precision scores, micro- and macro-average recall scores, accuracy rate, F1 score, and misclassification rate will be calculated. The most important metric is perhaps the F1 score, which is used to evaluate the performance of a classification model, such as XGBoost, by balancing precision and recall. It represents the harmonic mean of precision and recall, offering a single metric that encompasses both aspects of the model’s accuracy. When using models in the healthcare industry to make recommendations for diagnoses, the F1 score can be particularly helpful. A high F1 score suggests that the model is effective at identifying both positive and negative cases, which is crucial for reducing misdiagnosis and ensuring that patients receive the correct care [16].

Micro-average precision is the total of all the true positives divided by the total of all the true positives and false positives. In other words, it is the total number of accurate predictions divided by the total number of predictions. Macro-average precision is the arithmetic mean of the precision values for the different classes. The total of all the true positives for every class divided by the number of actual positives (as opposed to forecasted positives) yields the micro-average recall, and the macro-average recall is the arithmetic mean of all the recall scores for different classes [17]. 

One of the most straightforward performance metrics in machine learning is accuracy. It is a statistic that indicates how many of all the predictions were correct. This measure is quite simple to use in binary and multiclass classification problems, but it is important to understand its nuances and limitations. Accuracy is determined by dividing the total number of predictions by the sum of the true positives and true negatives. For example, a model would be 90% accurate if it made 100 predictions and 90 of them were correct (either true positives or true negatives). The misclassification rate is a machine-learning metric that represents the percentage of incorrect predictions made by a classification system [17].

## 3. Results

### 3.1. Model Training and Validation Results

Table 1 presents a comprehensive summary of the performance metrics for the XGBoost model across different histopathological diagnoses. The metrics include the F1 score, accuracy rate, misclassification rate, precision, and recall for both the training and validation datasets. 

As demonstrated in Table 1, the XGBoost model exhibits high accuracy and recall, indicating its effectiveness in identifying colorectal adenocarcinoma NOS, despite its lower precision suggesting some false positives. Validation data confirm the model’s strong performance on unseen data, maintaining high precision and recall with a minor trade-off in recall.

For a better representation and understanding of the findings, we grouped the diagnoses according to the model performance as follows: Group 1: high performance and generalization; Group 2: strong training performance, minor validation drops; Group 3: moderate performance with room for improvement; and Group 4: poor performance and generalization issues.

Group 1: High Performance and Generalization

- Prostatic acinar adenocarcinoma
- Gastric adenocarcinoma, NOS
- Pulmonary adenocarcinoma, NOS
- Basal cell carcinoma
- Invasive breast carcinoma, ductal type

These diagnoses show exceptional model performance, with high F1 scores, perfect or near-perfect recall, and high precision. The validation results indicate strong generalization to unseen data, suggesting robust and reliable model performance. For instance, the model’s perfect scores in training and very high validation scores for prostatic acinar adenocarcinoma and basal cell carcinoma highlight its robustness.

Group 2: Strong Training Performance, Minor Validation Drop

- Hepatocellular carcinoma
- Invasive breast carcinoma, lobular type
- Small cell neuroendocrine carcinoma
- Squamous cell carcinoma
- Serous carcinoma

These diagnoses exhibit strong training performance, with high F1 scores, perfect or high recall, and high precision. The validation results show slight decreases in precision or recall but maintain high performance overall. For example, the model’s training recall of 1.0000 for invasive breast carcinoma, lobular type, and its validation precision of 0.9091 indicate strong performance with minor validation drops.

Group 3: Moderate Performance with Room for Improvement

- Urothelial carcinoma
- Cholangiocarcinoma
- Glioblastoma
- Melanoma
- Kaposi sarcoma
- Undifferentiated pleomorphic sarcoma

These diagnoses show moderate performance, with high precision in training but significant drops in the recall and F1 scores in terms of validation, indicating difficulties in generalizing to unseen data. For instance, the model’s perfect precision for glioblastoma in validation contrasts with its low recall, highlighting the need for improvement in detecting true positives. Similarly, the model’s improved recall in validation for Kaposi sarcoma suggests better performance on new data.

Group 4: Poor Performance and Generalization Issues

- Colorectal mucinous adenocarcinoma
- Endometrioid carcinoma
- Invasive breast carcinoma, other types

These diagnoses exhibit poor performance, with low F1 scores and recall. The high precision in relation to training is misleading due to the very low recall, indicating many missed true cases. Validation metrics confirm this issue, showing significant decreases in precision and recall, suggesting difficulties in generalizing to unseen data. For example, the model’s low recall for colorectal mucinous adenocarcinoma and endometrioid carcinoma indicates it fails to identify many true cases. 

Overall, the XGBoost model’s high recall and accuracy across multiple carcinoma types make it a valuable tool for early and accurate cancer detection. Its ability to generalize well to new data ensures its reliability in real-world clinical settings. However, improving precision for certain carcinoma types can enhance performance, reducing false positives and unnecessary follow-up procedures. By addressing these areas through targeted improvements and following best practices for model deployment, the XGBoost model can significantly enhance diagnostic accuracy, providing robust support for clinicians in cancer diagnosis and treatment planning.

**Figure 2:**
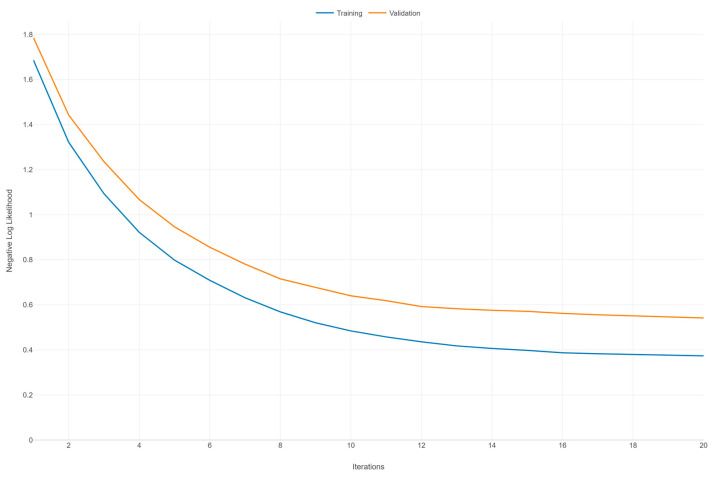
Learning curve of the XGBoost model: negative log likelihood vs. iterations.

Figure 2 illustrates the learning curves for an XGBoost model, depicting the negative log likelihood (NLL) against the number of iterations for both the training and validation datasets over 20 iterations. Both curves (training and validation) exhibit a general downward trend as the number of iterations increases. This indicates that the model is learning and improving its performance over time, with the NLL decreasing, which corresponds to better model predictions. The training NLL starts at around 1.6 and decreases steadily, flattening out to around 0.4 after 20 iterations. This consistent decrease signifies that the model is fitting the training data increasingly well. The validation NLL starts at around 1.8 and also decreases, but at a slower rate compared to the training curve. By the end of 20 iterations, it levels out around 0.6. The slower decrease compared to the training curve suggests that the model’s performance on unseen data is improving but not as rapidly as on the training data.

There is a noticeable gap between the training and validation NLL throughout the iterations, with the validation NLL consistently higher than the training NLL. This gap indicates that the model is fitting the training data better than the validation data, a common scenario that may suggest some degree of overfitting. However, since the validation NLL continues to decrease and does not show a marked increase or significant divergence, the overfitting is not severe. Both curves appear to be converging toward their respective minimums as the number of iterations increases, suggesting that additional iterations may not result in a substantial improvement in model performance.

### 3.2. Model Testing Results

After training and validating the model, a new set of 21 patients was used for testing. The following results were obtained after applying the model (Table 2). The model exhibits strong performance in predicting the following histopathologic diagnoses: squamous cell carcinoma, basal cell carcinoma, prostatic acinar adenocarcinoma, glioblastoma, invasive breast carcinoma ductal type, small cell neuroendocrine carcinoma, Kaposi sarcoma, and serous carcinoma. The precise alignment between the true labels and predicted labels for these diagnoses indicates the model’s high reliability and robustness, ensuring both high precision and recall. This consistency is crucial for clinical accuracy and minimizing the risk of misdiagnosis.

However, the model frequently misclassified pancreatic adenocarcinoma, gastric poorly cohesive carcinoma, pulmonary adenocarcinoma NOS, undifferentiated pleomorphic sarcoma, and endometrioid carcinoma into the “Other” category. This misclassification suggests that the model struggled to distinguish these specific cancer types, likely due to insufficient training data or overlapping features with other cancer types. For instance, pancreatic adenocarcinoma and undifferentiated pleomorphic sarcoma had few cases in the training set, resulting in inadequate learning by the model. Additionally, the misclassification of gastric poorly cohesive carcinoma as invasive breast carcinoma, lobular type indicates potential feature similarities or insufficient distinction in the IHC markers used.

The model’s high performance for several common cancer types indicates its potential value in clinical settings, aiding in early and accurate diagnosis, which is critical for effective treatment planning. However, the misclassifications and lower performance for certain rare or complex cancer types highlight the need for more comprehensive training data and the incorporation of additional diagnostic markers or advanced feature-engineering techniques to improve the model’s robustness.

Overall, the model shows promising results for many cancer types. Addressing its limitations through targeted improvements can enhance its diagnostic accuracy and reliability, thus providing better support for clinical decision-making. 

### 3.3. Model Performance Evaluation 

Table 3 presents a comprehensive performance summary of the XGBoost model, as evaluated on both training and validation datasets. The key metrics provided include the micro-average F score, macro-average F score, accuracy rate, and misclassification rate. These metrics collectively provide insights into the model’s predictive accuracy, generalizability, and reliability, which are critical for its application in clinical settings.

The micro-average F score aggregates the model’s performance across all instances to provide an overall measure of effectiveness. A score of 0.8597 for the training data indicates a high level of accuracy, reflecting the model’s ability to balance precision and recall. The validation score of 0.8137, though slightly lower, demonstrates strong performance and suggests that the model maintains a high level of predictive accuracy on unseen data. This minimal drop from training to validation suggests good generalization without significant overfitting.

The macro-average F score measures the model’s performance across all classes, treating each class equally. The training score of 0.8446 indicates consistent performance across different cancer types. The validation score of 0.8061, while slightly lower, still reflects robust performance on unseen data. This metric is particularly important for understanding the model’s ability to handle class imbalances and maintain performance across both common and rare cancer types.

The accuracy rate measures the proportion of correctly predicted instances. The high accuracy rate of 85.97% for the training data indicates that the model accurately classifies the majority of instances. The validation accuracy rate of 81.37%, though slightly lower, still reflects a high level of correctness on new data. The small decrease in accuracy from training to validation demonstrates the model’s reliability when applied to unseen data, which is critical for clinical applications.

The misclassification rate represents the proportion of incorrect predictions. A rate of 14.02% for the training data and 18.62% for the validation data highlights areas for improvement. The increased misclassification rate in the validation data suggests that while the model is effective, it encounters more challenges when predicting unseen cases.

Model Evaluation Summary:

- Strengths:
  ○ High precision and recall for several common cancer types, making it valuable for early and accurate diagnosis.
  ○ Consistent performance across different cancer types, essential for handling class imbalances in clinical datasets.
- Areas for Improvement:
  ○ The increased misclassification rate in the validation data indicates the need for more comprehensive training data, especially for rare cancer types.
  ○ Incorporating additional diagnostic markers or advanced feature-engineering techniques could improve the model’s ability to differentiate between similar cancer types, thereby reducing misclassification rates.

## 4. Discussion

### 4.1. State-of-the-Art Machine-Learning Algorithms for Histopathology

Traditionally, various machine-learning algorithms are applied to classification tasks, including Nearest Neighbor [18], Decision Trees [19], Artificial Neural Network [20], Support Vector Machine (SVM) [21], Ensemble Learning [22], and Convolutional Neural Network (CNN) [11]. These techniques aim to achieve high classification accuracy. However, they often struggle with challenges such as imbalanced misclassification costs across different classes. For example, these methods can have a higher likelihood of incorrectly identifying non-cancerous patients as cancerous or vice versa. This misclassification can lead to significant consequences, such as increased costs for further pathological examination. 

Regarding cancer detection and tumoral grading, Convolutional Neural Networks (CNNs) and Vision Transformers (ViTs) are extensively used for histopathological image analysis. For example, DINOv2 has shown exceptional performance in classifying colorectal cancer tissue patches [23]. These models excel in handling large-scale image data and performing automated feature extraction and representation learning. Chen et al. [24] introduced a hierarchical image pyramid transformer architecture that combines representations from multiple fields of view to obtain a slide-level representation, which is effective for processing gigapixel images in histopathology.

Another area of application of machine-learning models is in tumor segmentation and biomarker detection. Hierarchical self-supervised learning frameworks are used for precise segmentation of tumor regions within histopathological images, analyzing images at multiple scales to enhance accuracy [25]. Echle et al. [26] have utilized machine learning to quantify biomarkers like HER2 in breast cancer, achieving high performance in real-time clinical analysis. 

Several researchers have employed machine-learning models for feature extraction and survival prediction. For instance, Yao et al. [27] utilized attention-based multiple instance learning (MIL) networks for survival prediction by analyzing whole-slide images (WSIs). These networks focus attention on relevant patches, thereby enhancing the accuracy of survival predictions. Additionally, Hashimoto et al. [28] developed a multi-scale domain-adversarial convolutional neural network (CNN) for cancer subtype classification, utilizing unannotated histopathological images to mitigate performance variation across different sites. Other researchers have employed machine-learning models using IHC data to predict clinical outcomes in cancer patients. They compared the performance of these AI models with baseline features, establishing the superior efficacy of AI in predicting clinical outcomes [29,30].

XGBoost has been effectively combined with deep-learning models for tasks such as classifying breast tumors. For instance, combining DenseNet201 features with XGBoost has demonstrated high classification accuracy in breast cancer detection [25]. Also, XGBoost has been used to develop predictive models for high-risk conditions such as high-risk MASH (Maternal Alloimmune Hepatic Syndrome). The integration of SHAP (SHapley Additive exPlanations) enhances the interpretability of these models, which is crucial for clinical applications [31].

It can be noticed that the XGBoost model has not yet been explored for classifying a variety of histopathological diagnoses based on particular immunohistochemical markers.

### 4.2. Performance Metrics and Generalization

In the present study, we validated the estimated diagnostic probability of specific histopathological diagnoses based on particular immunohistochemical markers. This was achieved using a machine-learning system employing a probabilistic Decision Tree model, such as XGBoost. These models have been applied to identify lung cancer and colon cancer subtypes [32], predict lung metastases from thyroid cancer [33], and develop risk models for detecting lung cancer [34], all of which have demonstrated high performance.

Our study demonstrated a precision rate of 85.97% within the dataset, indicating a high level of performance and suggesting that the model is generally reliable in producing accurate predictions. The precision rate of the predicted diagnoses was generally higher compared to prior research on tumors of unknown origin, which reported a precision rate of 79.9% [35]. However, it was lower than a study using lymphoma cases, which achieved a precision rate of 95% [36]. 

A high precision rate was observed in diagnoses such as adenocarcinoma with various localizations and melanoma. The authors attributed these findings to the fact that the machine-learning system provides more accurate predictions when trained on a larger number of cases. Other studies have shown that the accuracy rate for each tumor type is closely related to the corresponding sample size. Moreover, an imbalance in the dataset complicates the achievement of high performance [37,38,39].

Trying to understand the issues that led to cases being classified under a different diagnosis than the correct one, we analyzed each case in the testing set individually. In the case of pancreatic adenocarcinoma classified as “Other”, the issue is that the training set included only six cases with this diagnosis, which was not sufficient for learning and creating a distinct class, as this diagnosis was not among the 20 subtypes mentioned in Table 1. A similar explanation can be applied to undifferentiated pleomorphic sarcoma, which had five cases in the training set. For poorly cohesive gastric carcinoma, the training set had only three cases, which did not allow for the creation of a class. However, due to the use of common markers for both diagnoses (CK7, HER2, E-cadherin), the model classified it as lobular breast carcinoma instead of labeling it as “Other”. The case of lung adenocarcinoma classified as “Other” is atypical; as there were sufficient data in the training set, the F1 score was quite high and the precision was 1.0000. The explanation for this could be that it was a poorly differentiated brain metastasis, requiring the use of a larger number of markers (specifically 11) to establish the diagnosis, which likely led to an inaccurate prediction. The situation where an endometrioid carcinoma had the predicted label “Other” is attributed to the fact that this class has an F1 score of 0.7778, indicating moderate performance, most likely due to the lack of specificity of the IHC markers for this tumor subtype. The challenge of imbalanced data is critical in histopathology, where rare cancer types are often underrepresented. This study’s results align with the broader literature emphasizing the need for comprehensive and balanced datasets to enhance model accuracy [25,31]. While the model showed high performance for common cancer types, its generalization to rare types was limited. This is consistent with the challenges faced by other state-of-the-art models, suggesting improvements in training data diversity and advanced feature-engineering are necessary to enhance generalization capabilities [23,31].

As aforementioned, the present study employed a binary tumor marker expression system (positive or negative) to forecast the histopathological diagnosis. Nevertheless, alternative investigations have focused on developing mathematical models capable of predicting cancer types using molecular characteristics, such as gene expression, DNA methylation profiles, or somatic alteration analysis [40,41,42,43]. Through the inclusion of driver gene mutations and complex structural variant-related features, Nguyen et al. [43] achieved the prediction of approximately 35 different cancer (sub)types with an accuracy of approximately 90%. Marquard et al. [44] successfully distinguished between 10 cancer types with 69% accuracy and 6 cancer types with 85% accuracy when considering solely somatic mutations or both somatic mutations and copy number alterations, respectively. Utilizing the presence of somatic point mutations and copy number alterations in 50 genes as predictive features, Soh et al. [38] attained an accuracy of approximately 77% across 28 distinct cancer types. 

Another method for predicting histopathological diagnoses, documented in the scientific literature, involves employing machine-learning systems that analyze cellular properties like nuclear size and the homogeneity of cell populations. This approach holds clinical utility for distinguishing various cell groups. Moran et al. [45] utilized the cell detection module within QuPath, an open-source image analysis toolbox for digital pathology, to extract morphological features of Merkel cell carcinoma (MCC). Their findings indicated that the nuclear area and circularity were significant prognostic factors for MCC. Faridi et al. [46] employed region-growing methods to segment cells, extracting morphological features such as the nuclear size, solidity, and eccentricity to grade the pleomorphism scores of whole-slide images of breast cancer cases, and attained an accuracy of 86.6%.

### 4.3. Challenges, Limitations, and Recommendations for Future Research

The model frequently misclassified certain rare or complex cancer types. This misclassification indicates that the model struggled to differentiate these specific cancer types, likely due to insufficient training data or overlapping features with other cancer types. For example, pancreatic adenocarcinoma and poorly cohesive gastric carcinoma were often misclassified, highlighting the need for more comprehensive training data and advanced feature-engineering techniques to enhance the model robustness. This challenge aligns with the findings from other studies that emphasize the necessity for more extensive training data and sophisticated feature engineering to improve the model performance [23,35,36,37,38,39].

While the model demonstrated high performance for several common cancer types, its performance dropped when applied to rare or less common types. This indicates a limitation in the model’s ability to generalize across all cancer types. This study suggests that increasing the sample size for each diagnostic category and incorporating additional diagnostic markers could enhance the model’s generalization capabilities. This recommendation is consistent with other research that advocates for techniques like regularization to improve generalization [25,35,42].

This study notes that there was an imbalance in the dataset used for training the machine-learning model. Certain tumor types had significantly fewer cases, which affected the model’s ability to learn and accurately predict these diagnoses. For instance, the training set included only a small number of cases of pancreatic adenocarcinoma and undifferentiated pleomorphic sarcoma, making it difficult for the model to create distinct classes for these diagnoses. Increasing the sample size for each cancer type and incorporating additional diagnostic markers can improve the model’s robustness and accuracy [16,17,31].

To enhance the model’s learning and prediction capabilities, we could expand the dataset to include more cases for each cancer type, with a particular focus on rare ones. Additionally, incorporating a greater variety of diagnostic markers and employing advanced feature-engineering techniques could improve differentiation between similar cancer types. Exploring the use of more complex data systems, such as gene expression and DNA methylation profiles, would further capture the intricacies of tumor marker expressions. 

## 5. Conclusions

This study demonstrated the feasibility and efficacy of utilizing the probabilistic Decision Tree algorithm to differentiate tumor diagnoses according to immunohistochemistry profiles. 

The primary sources of error within this system include disease-specific markers, the presence of overlapping IHC profiles among diseases, a deficiency of site-specific markers, mixed or combined tumors, and atypical IHC profiles. Another source of error arises from the limited number of cases available in the training dataset for each diagnosis. Future research should address this by increasing the sample size for each diagnostic category, allowing for more robust analysis and validation of the findings. Nevertheless, this system can serve as a valuable aid to pathologists in rendering accurate diagnoses. 

## Figures and Tables

**Table 1:** XGBoost learning model summary by class.

Class		F1 Score	Accuracy Rate	Misclass. Rate	Precision	Recall
Colorectal adenocarcinoma, NOS	Training	0.8333	0.9819	0.0181	0.7143	1.0000
	Validation	0.8235	0.9705	0.0295	0.7368	0.9333
Colorectal adenocarcinoma, mucinous	Training	0.3333	0.9819	0.0181	1.0000	0.2000
	Validation	0.2102	0.8921	0.1079	0.3478	0.5333
Prostatic acinar adenocarcinoma	Training	1.0000	1.0000	0.0000	1.0000	1.0000
	Validation	0.9231	0.9950	0.0050	1.0000	0.8572
Gastric adenocarcinoma, NOS	Training	0.9091	0.9910	0.0090	0.8333	1.0000
	Validation	0.8889	0.9958	0.0042	0.8000	1.0000
Pulmonary adenocarcinoma, NOS	Training	0.8889	0.9910	0.0090	1.0000	0.8000
	Validation	0.9090	0.9852	0.0148	0.8333	1.0000
Basal cell carcinoma	Training	0.9524	0.9955	0.0045	0.9091	1.0000
	Validation	1.0000	1.0000	0.0000	1.0000	1.0000
Endometrioid carcinoma	Training	0.7778	0.9819	0.0181	0.8750	0.7000
	Validation	0.5454	0.9755	0.0245	0.6000	0.5000
Hepatocellular carcinoma	Training	1.0000	1.0000	0.0000	1.0000	1.0000
	Validation	0.9230	0.9902	0.0098	1.0000	0.8571
Invasive breast carcinoma, other types	Training	0.5714	0.9729	0.0271	1.0000	0.4000
	Validation	0.5445	0.9792	0.0208	0.6000	0.5000
Invasive breast carcinoma, ductal type	Training	0.8333	0.9819	0.0181	0.7143	1.0000
	Validation	0.9217	0.9626	0.0374	0.8689	0.9815
Invasive breast carcinoma, lobular type	Training	0.9524	0.9955	0.0045	0.9091	1.0000
	Validation	0.9333	0.9951	0.0049	0.8750	1.0000
Small cell neuroendocrine carcinoma	Training	0.8000	0.9819	0.0181	0.8000	0.8000
	Validation	0.7714	0.9876	0.0124	0.6667	0.3333
Squamous cell carcinoma	Training	0.9474	0.9955	0.0045	1.0000	0.9000
	Validation	0.9523	0.9853	0.0147	0.9091	1.0000
Serous carcinoma	Training	0.9474	0.9955	0.0045	1.0000	0.9000
	Validation	0.9523	0.9853	0.0147	0.9091	1.0000
Urothelial carcinoma	Training	0.8571	0.9864	0.0136	0.8182	0.9000
	Validation	0.7778	0.9804	0.0196	0.7000	0.8750
Cholangiocarcinoma	Training	0.8000	0.9910	0.0090	1.0000	0.6667
	Validation	0.7714	0.9884	0.0116	0.6667	0.3333
Glioblastoma	Training	0.8696	0.9864	0.0136	0.7692	1.0000
	Validation	0.6667	0.9951	0.0049	1.0000	0.5000
Melanoma	Training	1.0000	1.0000	0.0000	1.0000	1.0000
	Validation	0.9231	0.9951	0.0049	1.0000	0.8571
Kaposi sarcoma	Training	0.8889	0.9955	0.0045	1.0000	0.8000
	Validation	0.8788	0.9833	0.0167	0.8788	0.8788
Undifferentiated pleomorphic sarcoma	Training	0.7500	0.9910	0.0090	1.0000	0.6000
	Validation	0.5455	0.9755	0.0245	0.6000	0.5000
Other	Training	0.8247	0.9231	0.0769	0.7843	0.8696
	Validation	0.8235	0.9706	0.0294	0.7368	0.9333

**Table 2:** Test dataset results.

Histopathologic Diagnosis	Predicted Label
Squamous cell carcinoma	Squamous cell carcinoma
Basal cell carcinoma	Basal cell carcinoma
Prostatic acinar adenocarcinoma	Prostatic acinar adenocarcinoma
Glioblastoma	Glioblastoma
Invasive breast carcinoma, ductal type	Invasive breast carcinoma, ductal type
Squamous cell carcinoma	Squamous cell carcinoma
Squamous cell carcinoma	Squamous cell carcinoma
Pancreatic adenocarcinoma	Other
Gastric poorly cohesive carcinoma	Invasive breast carcinoma, lobular type
Pulmonary adenocarcinoma, NOS	Other
Undifferentiated pleomorphic sarcoma	Other
Small cell neuroendocrine carcinoma	Small cell neuroendocrine carcinoma
Invasive breast carcinoma, ductal type	Invasive breast carcinoma, ductal type
Invasive breast carcinoma, ductal type	Invasive breast carcinoma, ductal type
Kaposi sarcoma	Kaposi sarcoma
Small cell neuroendocrine carcinoma	Small cell neuroendocrine carcinoma
Pulmonary adenocarcinoma, NOS	Pulmonary adenocarcinoma, NOS
Prostatic acinar adenocarcinoma	Prostatic acinar adenocarcinoma
Small cell neuroendocrine carcinoma	Small cell neuroendocrine carcinoma
Serous carcinoma	Serous carcinoma
Endometrioid carcinoma	Other

**Table 3:** Model performance summary.

Data Type	Micro-Average F Score	Macro-Average F Score	Accuracy Rate	Misclass. Rate	Rows
Training	0.8597	0.8446	0.8597	0.1402	481
Validation	0.8137	0.8061	0.8137	0.1862	204

## Data Availability

The original contributions presented in the study are included in the article, further inquiries can be directed to the corresponding authors.
